# Influence of In Situ Polymerization on the Compressive Strength of Scots Pine (*Pinus sylvestris* L.) Recovered from Demolition Timber and Two Forest-Sourced Species: European Beech (*Fagus sylvatica*) and Black Alder (*Alnus glutinosa*)

**DOI:** 10.3390/ma18153439

**Published:** 2025-07-22

**Authors:** Emil Żmuda, Kamil Roman

**Affiliations:** 1 Warsaw University of Life Sciences—SGGW, Department of Wood Science and Wood Protection, 159 Nowoursynowska, 02-787 Warszawa, Poland; emil_zmuda@sggw.edu.pl; 2 Warsaw University of Life Sciences—SGGW, Institute of Wood Sciences and Furniture, Department of Technology and Entrepreneurship in the Wood Industry, 159 Nowoursynowska, 02-787 Warszawa, Poland

**Keywords:** wood-plastic composites, polymer, plastic, wood, in-situ modification

## Abstract

This study investigated the effect of in situ polymerization on the compressive strength of demolition-derived Scots pine, European beech, and black alder wood. The treatment applied was based on previously confirmed in situ polymerization systems in wood, which are known to lead to polymer formation and composite-like structures. In this study, we assumed similar behavior and focused on a mechanical evaluation of the modified wood. Three different polymer systems were applied to evaluate differences in performance. After modification, the compressive strength levels increased by 60% in beech, 119% in alder, and 150% in pine, with corresponding increases in density and weight percent gain (WPG). The highest relative improvement was observed in the least dense species, pine. The findings suggest that polymer treatment can significantly enhance the mechanical properties, likely due to the incorporation of polymer into the wood matrix; however, this inference is based on indirect physical evidence.

## 1. Introduction

Wood is one of the oldest and most versatile materials, with widespread applications in construction, furniture production, and countless other fields. Its natural origin, renewability, and biodegradability make it an environmentally friendly material, a characteristic growing in importance with our increasing environmental awareness [1]. Wood is readily available and relatively affordable, making it an attractive raw material for many industries. In construction, particularly for structures, material strength is crucial [2]. Wood boasts a good strength-to-weight ratio and is relatively lightweight yet strong under tension, compression, and bending. Combined with its ease of processing, wood is ideal for various structures, from small objects to large buildings. Wood has inherent aesthetic qualities; its natural appearance, warm color, and diverse grain patterns give wood products a unique character [3]. The absence of chemical characterization studies of polymers formed in wood was a significant limitation of this study. Currently, there is no information on the polymer identity, crosslinking degree, or cell wall bonding efficiency, so it is impossible to fully understand the differences between the three modification types. Physical and mechanical tests offer comparative insights but may not fully explain chemical interactions. According to the literature reports, the performance of wood–polymer composites can be influenced significantly by variations in monomer ratios or polymerization conditions [4].

Wood also has its drawbacks. It is a combustible material, which poses a danger in case of fire. Using treated wood or thick sections can make it more fire-resistant, even though it is flammable. Large timber beams can resist damage during a fire and remain structurally sound for extended periods, providing valuable time for evacuation and firefighting. In addition, it is susceptible to moisture, fungi, and insects, which can cause it to decay and deteriorate. To retain its properties, wood must be adequately protected against these factors, such as via impregnation or painting. Another disadvantage of wood is its dimensional variability under changes in temperature and humidity [5]. Wood can swell or shrink, which can lead to cracks and deformations. Wood cracking is caused by the uneven drying of different wood parts, leading to internal stresses. The low resistance of wood to abrasion and impact may limit its use in some areas. Additional protection, such as with a varnish or impregnation, is necessary for wood exposed to weathering, such as from rain or sunlight. Despite these drawbacks, wood remains a valuable and popular material, and its properties can be improved with proper treatment and protection [6,7]. Numerous previous studies have confirmed that such in situ polymerization processes result in polymer formation and distribution within the wood matrix, verified through FTIR, SEM, and TGA analyses [8,9]. Since our experimental conditions closely followed these validated protocols, we focused on a mechanical evaluation rather than repeating such chemical confirmation approaches.

This study investigated the impact of polymerization on the mechanical properties of three wood species—Scots pine, European beech, and black alder. Wood specimens were impregnated with polymerizing monomers, and their compressive strength was measured to assess the structural improvements. This article is structured as follows. Section 2 describes the wood selection process, polymerization method, and mechanical testing procedures. Section 3 presents the results, highlighting changes in compressive strength and stiffness due to polymerization. Section 4 compares these findings with the existing research, analyzing factors such as the wood anatomy and polymer distribution that influence the modification’s effectiveness. Finally, Section 5 summarizes the main conclusions and suggests future research directions, including long-term durability assessments, environmental considerations, and further optimization of the polymerization process.

The benefits of wood and plastic can often be combined to overcome some of the disadvantages. Wood–polymer composites (WPC) [10] are an example of this. Wood–plastic composites have enhanced resistance to moisture, fungi, and insects while maintaining a natural appearance. The polymers used to create composites can also increase the plasticity of wood, making it easier to mold and process and improving its structure, making it more resistant to mechanical damage [11]. Modifying polymers can also enhance the durability of wood against weathering conditions, such as UV rays and high temperatures. The literature contains examples of polymer-modified timber used in various applications [12,13,14]. For instance, polymers have been added to pine wood to increase its flexural strength significantly. The material can also make weather-resistant facade panels, durable garden furniture, and structural elements with increased load-bearing capacity [15].

Despite the advantages, WPCs may have some disadvantages compared to natural wood, such as using non-renewable materials and having the potential for worse fire performance depending on the polymer. Certain polymers used in WPCs may release toxic fumes when burned, which can be hazardous to health. The modification of wood, often involving recycled polymers, can enhance its strength and resistance to mechanical damage and weathering. It also reduces the amount of waste going to landfills [16,17]. Wood modified polymers can be sued to produce durable and moisture-resistant garden furniture, moisture-resistant building facades, and even structural elements with increased load-bearing capacity. Researchers have shown that modifications significantly increase pine wood’s flexural strength [18,19], opening new prospects for using modified timber in construction. The use of WPCs leads to more efficient use of natural resources and reductions in environmental damage.

The wood industry is embracing the sustainable use and production of wood–plastic composites (WPCs) [20]. Using polymer waste to make WPCs can reduce the waste going to landfills [21]. Polymer waste can be used in the polymerization process to produce sustainable materials, and this study will explore the feasibility of this approach by using recycled polymers in the modification process. Recycled polymers can achieve comparable or superior mechanical properties to virgin polymers while reducing their environmental impact. Wood polymerization is an innovative method for producing WPCs that differs from conventional methods by directly introducing monomers into wood structures, thereby improving the properties of the composite. Wood polymerization occurs in situ when monomers are introduced directly into wood structures, resulting in composite materials with enhanced properties. The process differs from traditional WPC production, which blends wood fibers and polymers. This article discusses the wood polymerization method, which differs from traditional wood and plastic production methods by introducing monomers directly into the wood structure, enabling better property integration between the polymer and wood matrix [10]. Polymerizing wood involves introducing monomers directly into its structure, which allows the two materials to combine and improve their properties, creating a composite with unique characteristics.

This study addresses a critical gap in the wood modification research by evaluating the effect of in situ polymerization on the compressive strength of reclaimed demolition timber. While prior studies have focused predominantly on bending properties, the compressive strength—especially in aged, reused wood—remains underexplored, despite its importance for structural applications. The novelty of this work lies in comparing three distinct polymer formulations and their effects across three wood species, including aged pine recovered from structural elements. By using in situ polymerization instead of traditional blending methods, this study introduces a direct modification technique that allows deeper polymer penetration into the wood matrix. Through an analysis of the compressive strength, density, and weight percentage gain (WPG), the aim of this study was to optimize polymer–wood interactions for performance enhancements and contribute to the sustainable reuse of timber in construction.

## 2. Materials and Methods

### 2.1. Materials

#### 2.1.1. Material Characteristics

This study employed three distinct wood species—Scots pine (*Pinus sylvestris* L.), European beech (*Fagus sylvatica*), and black alder (*Alnus glutinosa*). Ten samples were prepared for each type of wood. The Scots pine (*Pinus sylvestris*) was uniquely sourced from the structural elements of an approximately 80-year-old building, allowing an investigation of the modification of aged wood. The old pine material was reclaimed from roof truss elements of an 80-year-old residential building. These structural components were likely exposed to long-term mechanical loads and environmental conditions, which may have affected their internal structure and strength properties. In contrast, the beech and alder samples were obtained from the Mazovia Forest District in Poland. The selection of pine for this research was due to its prevalence in diverse applications, including furniture, structural components, and wood-based panels. Its widespread use can be attributed to several favorable characteristics, such as its ease of processing, relatively high compressive strength, cost-effectiveness, and availability. It is essential to recognize that inherent variations arising from natural growth patterns exist within each wood species. In the case of the aged pine, these variations may have been further amplified by prolonged exposure to environmental factors during its service life. Factors such as the type, strength, knot frequency and size, resin content, and hygroscopicity can significantly influence the wood’s behavior during the polymerization process, ultimately affecting the quality and performance of the resultant wood–polymer composite [20].

The polymerization of solid wood offers the potential to enhance its mechanical properties, notably its strength, bending resistance, and compressive strength, while concurrently improving its resistance to biological and atmospheric degradation. Ten samples were collected from each species across three polymer modification variants and a control group. Based on a factorial approach, we assessed how different wood types and polymerization mixtures affect the mechanical and physical properties. It is important to note that the initiator and activator concentrations varied between the three polymer mixtures. Formulations were selected to evaluate how different polymer compositions influence the mechanical performance, particularly the compressive strength and stiffness. The chemistry of the mixture can impact the distribution of polymers within the wood, as well as the bonding of polymers to the cell walls of the wood.

The three wood species tested in this study have distinct chemical compositions, which may impact their properties and behavior during polymerization. The Scots pine (*Pinus sylvestris*) possesses a high level of lignin and cellulose, with lower levels of hemicelluloses. The lignin in pine contains primarily guaiacyl units that contribute to its decay resistance. The European beech (*Fagus sylvatica*) is a hardwood with a high proportion of hemicelluloses. Guaiacol and syringyl units in the beech lignin may influence its polymerization reaction. It has a chemical composition similar to beech but contains more extractives than beech. In modifying wood, these extractives can influence its permeability and reactivity. Differences in chemical composition may contribute to observed changes in moisture levels. Alder, for example, has a higher extractive content than beech and pine, contributing to its higher moisture content. Lignin can also influence the wood’s hygroscopicity or ability to absorb and retain moisture.

This study used styrene (MY-CHEM GmbH, Innungsstraße 11, 21244 Buchholz i.d. Nordheide, Germany) as the main component of the monomers with 2% or 3% maleic anhydride (Thermo Scientific, 5781 Van Allen Way, Carlsbad, CA, USA, manufactured in Gosselies, Belgium), an ingredient that is designed to increase the bonding of the polymer to the cell wall, as well as an initiator, 1% benzoyl peroxide (Sigma-Aldrich, Merck KGaA, Darmstadt, Germany; EMD Millipore Drive Corporation 400 Summit Drive, Burlington, MA, USA, made in Germany), which is needed to initiate the polymerization of styrene. The styrene monomer and other components in this study were not recycled, due to the lack of specialized equipment needed to obtain such materials. The following equipment and materials were used in the study, with all devices regularly calibrated:

- Analytical balance: PS 600/C/2 (Radwag, Radom, Poland), resolution 0.01 g—used for precise mass measurements before and after polymerization.
- Laboratory dryer: SLW 115 STD TOP+ (POL-EKO, Ostrów Wielkopolski, Poland), temperature range up to 250 °C—used for both pre-conditioning of samples and polymerization curing.
- Vacuum chamber: Custom-made borosilicate glass chamber equipped with a top-mounted analog vacuometer (±5 mbar accuracy), stainless steel inlet and outlet valves, and tubing connectors—used to evacuate air from the wood prior to monomer saturation.
- Digital caliper: CDN-NT Bluetooth (Limit, Luna Polska, Dębica, Poland), resolution 0.01 mm—used for dimensional measurements of samples pre- and post-treatment.
- Vacuum pump: Oil rotary vane pump (KNF Neuberger, Maulburg, Germany, model N 840.3 FT.18), final pressure ~10 mbar—used for creating a vacuum in the chamber.
- Conditioning chamber: Climate-controlled chamber with saturated salt solution (Mg(NO_3_)_2_) for ~50% RH at 23 °C—used to stabilize the samples before testing.
- Testing machine: Instron 3382 (Instron, Norwood, MA, USA), load capacity of 50 kN, equipped with InstronX software—used for compression testing in accordance with ASTM D143-14.

#### 2.1.2. Moisture Content

In order to stabilize their moisture content, all wood samples were conditioned at room temperature and ambient humidity for approximately one year prior to polymerization. The moisture content of representative specimens from each wood species was measured using the oven-dry method. Using this technique ensured consistent conditions across experimental groups and minimized the effect of moisture variability on the polymer uptake and mechanical testing.

### 2.2. Pre-Treatment Sample Procedure

This study used three different wood species to modify the lignocellulosic raw materials. The use of post-collapse pine provided a unique opportunity to assess the modification potential of aged wood. Before modification, samples from each species were cut into dimensions of 30 × 20 × 20 mm. The samples in the study were measured using a digital caliper. To ensure consistency and reproducibility, the initial moisture content of each wood species was determined using the oven-dry method. The results revealed the following moisture content values, namely 8.97% ± 0.41% for beech, 9.76% ± 0.29% for alder, and 9.64% ± 0.24% for pine. Moisture content measurements were carried out using the dryer–weigher method using a top+ dryer (POL-EKO, Ostrów Wielkopolski, Poland) and a Radwag balance (Radom, Poland) on samples not involved in this study but coming from the same batch of wood. Although relatively small, these differences in initial moisture content highlight the inherent variability even within wood species. For each study group, 10 samples were used per modification type, including the control group, resulting in 40 samples per species and 120 samples in total.

The proportions of styrene, maleic anhydride, and benzoyl peroxide varied between the three formulations of polymers but no chemical analysis was performed to determine their exact compositions. This study solely compared physical and mechanical characteristics. The limitations of this study are acknowledged, and future studies should include structural and spectroscopic analyses (e.g., FTIR, SEM, or TGA) to more deeply understand the chemistry of the resulting polymer–wood composites. Styrene (MY-CHEM GmbH, Innungsstraße 11, 21244 Buchholz i.d. Nordheide, Germany) with 2% or 3% maleic anhydride (Thermo Scientific, 5781 Van Allen Way, Carlsbad, CA, USA, manufactured in Belgium) and an initiator, 1% benzoyl peroxide (Sigma-Aldrich, Merck KGaA, Darmstadt, Germany; EMD Millipore Drive Corporation 400 Summit Drive, Burlington, MA, USA, manufactured in Germany), were used in this research.

Despite the lack of chemical characterization of polymer formulations in the study, the formulations were chosen on the basis of the literature indicating that maleic anhydride can bind to lignin and cellulose in wood, improving its mechanical strength and polymer retention [22,23]. The free-radical initiator benzoyl peroxide promotes styrene polymerization, while the coupling agent maleic anhydride enhances the adhesion between hydrophobic polymers and hydrophilic wood walls. Based on prior research, we include 2% and 3% maleic anhydride contents because these concentrations optimize the grafting efficiency without causing excessive crosslinking or brittleness. FTIR and SEM analyses will be performed in future studies to confirm the chemical bonding and polymer distribution, even though no spectroscopic verification was performed in this study. Maleic anhydride is reported to enhance polymer retention and strength properties by promoting covalent bonding between styrene and wood cell wall components, especially in lignin [3,24]. In order to examine whether controlled compositional changes could improve the interactions between wood and polymer, we varied the maleic anhydride contents across the three formulations (0%, 2%, and 3%). Increasing activator levels are associated with stronger polymer integration, as evidenced by increased WPG and compressive strength values. Future work should involve FTIR or SEM imaging to directly verify the presence and distribution of polymers in the wood matrix.

Our in situ modification process used a specific saturation procedure to ensure adequate penetration of the monomer mixture into the wood structure. In this process, the samples were initially selected, weighed, and measured to determine their dimensions. To ensure homogeneous distribution and prevent premature polymerization, the activator (maleic anhydride) and initiator (benzoyl peroxide) were thoroughly mixed with the styrene monomer before immersion. Subsequently, the samples were submerged in the monomer mixture within containers. A suitable weight was placed on the samples within each container to prevent sample flotation and ensure complete immersion. The samples were placed at a small distance from the bottom and at distances between each other. In addition, the samples lay on one of the side surfaces to allow the monomer to penetrate through the face.

In order to prepare the samples, they were first submerged in the monomer mixture and placed in beakers. A vacuum chamber was then used to transfer these containers. Rather than removing the monomers, the vacuum process removed air from the porous structure of the wood. During the extraction process, the vacuum pump and chamber were evacuated to about 100 mbar and maintained for 30 min, and the measurement was observed on an analog vacuometer. It was necessary to repeat this vacuum–release cycle twice to ensure that the monomer penetrated the wood structure more deeply and uniformly. With this saturation protocol, utilizing a cyclic vacuum application, the modifying agents penetrated deeper into the complex porous structure of the wood and were distributed more efficiently, especially for species such as beech and aged pine, which may be resistant to impregnation. The compositions of the polymerization mixtures are presented in Table 1.

The styrene used above was of technical purity. Benzoyl peroxide at approximately 1% was added to initiate styrene polymerization, and a maleic anhydride activator at roughly 2% was added to bind the styrene to the wood cell wall. The samples were carefully removed from the monomer solution following the saturation process. The surfaces were meticulously dried with absorbent paper to prevent the monomer from leaching. Two layers of polyethylene film were then used to wrap each sample before it was heat-sealed to create an air-tight environment. The purpose of this encapsulation was twofold: firstly, to prevent the evaporation of volatile monomers during the curing process; secondly, to minimize the exposure to atmospheric oxygen, which could inhibit polymerization. The samples prepared in this manner were subjected to a staged heat treatment to initiate and complete the polymerization process. The samples were placed in a laboratory dryer preheated to 80 °C for 24 h. To minimize thermal stress and defect formation in wood structures, a lower initial temperature was chosen to allow for more gradual and controlled initiation of polymerization. To ensure complete crosslinking and curing of the monomer, the temperature was increased to 105 °C for another 24 h to maximize the composite’s mechanical strength and dimensional stability. Following the heat treatment, the samples were unwrapped from the foil and placed loosely in the dryer for a minimum of 24 h at 105 °C. Any remaining unreacted monomer or volatile byproducts formed during curing were removed during the final drying step. Finally, the samples were accurately measured and weighed. The measurements allowed for the calculation of the density of the modified wood and the determination of the weight percent gain (WPG) parameter, which indicates the extent of monomer uptake.

### 2.3. Strength Tests

#### 2.3.1. Straight Sample Preparation

The compressive strength of the modified wood was determined by conducting tests parallel to the grain. This study utilized specimens of pine, beech, and alder. Each sample was prepared in a cuboidal shape, and the moisture content was standardized before testing using a conditioning chamber [25]. Testing was conducted on all samples that had a moisture content of 12%. The sample preparation and testing followed ASTM D143-14 Standard Test Methods for Small Clear Specimens of Timber [26]. Standard laboratory conditions were used to conduct all tests on an Instron 3382 universal testing machine (50 kN capacity). During the compression test, constant compressive force was applied along the longitudinal axis of the specimens. The compression tests were conducted at a speed of 5 mm/min along the longitudinal axis. The observations were in accordance with ASTM D143-14 for the loading rate. The static compression test was concluded when the specimen exhibited noticeable deformation or failed. The samples were terminated after losing 5% of their original height or clearly breaking.

#### 2.3.2. Procedure of Strength Tests

The compression tests were conducted according to ASTM D143-14 [26] on a test machine. Constant compressive force was applied along the longitudinal axis of the specimens at a steady speed of 5 mm/min. The test measures the maximum force the material can withstand before failure, providing data to calculate its compressive strength and stiffness. In the context of this study, it was hypothesized that the polymerization process used for wood modification would enhance the ductility of the tested samples. The compressive strength parallel to the grain was calculated by dividing the maximum destructive force, as recorded by the force gauge, by the cross-sectional area of the specimen perpendicular to the force application. Using these measurements, the study aimed to compare the mechanical performances of untreated and modified samples. The testing conditions were controlled in order to minimize variation and ensure repeatability.

### 2.4. Statistical Analysis

Statistical analyses are crucial in research, identifying key factors influencing observed results and determining significant differences between experimental groups [27]. A foundational concept in such analyses is variance, which quantifies the spread or dispersion of data points around the mean. By employing a variance analysis, researchers can assess the distribution and variability of their data to determine the consistency and reliability of their findings. An analysis of variance (ANOVA) is a widely used statistical technique, particularly in studies involving more than two groups [28,29]. An ANOVA facilitates the simultaneous comparison of multiple groups, accounting for both within-group and between-group variability. The fundamental principle of an ANOVA involves comparing the variance observed between groups to the variance within groups. When the variance between groups significantly exceeds the variance within groups, it suggests that the observed differences are likely attributable to the experimental treatment or factor under investigation rather than random chance. This ability to discern the influence of specific factors is essential for researchers to draw meaningful conclusions from their data.

## 3. Results

### 3.1. Analisis of Material Characteristics

#### 3.1.1. Moisture Measurement

The moisture content was measured before polymerization for all wood species to establish baseline conditions. These values were used to contextualize later changes in physical and mechanical properties. The moisture contents of the materials before polymerization are described in Table 2.

Before the polymerization process began, a moisture analysis was performed on the wood samples to determine the moisture level. Every species was tested to determine its average moisture content, expressed as a percentage. To interpret the variability within each group, standard deviation values were calculated. The ANOVA revealed a statistically significant effect of the wood species on their initial moisture content. We supplemented the statistical analysis with Duncan’s post hoc test. The *F*-statistic was 17.67, with 2 and 27 degrees of freedom, and the *p*-value was less than 0.00001, indicating strong evidence for differences between the compared groups. This implies that the examined groups, representing different wood species, exhibited statistically significant differences in moisture levels. Before polymerization, the moisture content was measured only for the overall batch of each species, since the modification was not expected to affect initial moisture. Including reference and modified subgroups could provide additional insights, which will be considered in future research studies. Differences in moisture content can be attributed to the wood species’ chemical composition, including the amount of extractive content, lignin types, and amounts of lignin, which affect the wood’s hygroscopicity.

#### 3.1.2. Physical Property Measurements

Before polymerization, all wood samples were measured for their length, width, thickness, and mass using calibrated instruments to calculate their density. The samples were randomly assigned to either the reference group or one of three modification groups before any chemical treatment was applied. Although the physical measurements were conducted prior to polymerization, grouping by assigned modification type was used to ensure comparability with the post-treatment results. The results are shown in Table 3.

The group labels (modifications 1–3) reflect the pre-assigned treatment groups; all measurements were taken before polymerization. Three types of modifications were included in the samples, which were measured, referenced, and modified. The average length, width, thickness, and weight of each sample were calculated, as well as the standard deviation. Based on these data, modification-induced changes can be analyzed, and the material’s original state can be illustrated. It was evident from the initial measurements that there were clear differences in the average densities between species. The old pine and beech showed the highest density values, followed by the alder and beech. The differences can be attributed to the anatomical characteristics of the wood species.

**Figure 1:**
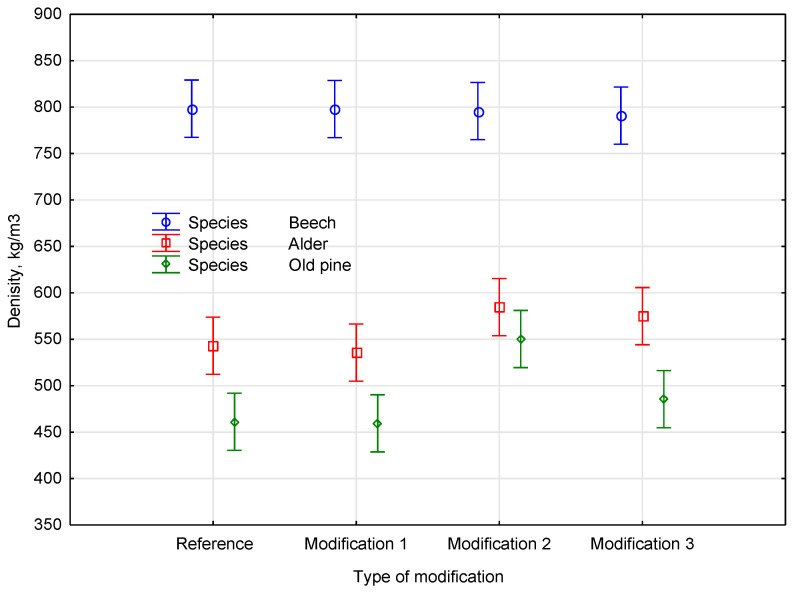
The impact of the material density before polymerization by species and assigned modification group (prior to treatment).

Softwoods such as pine are composed mainly of tracheids, with limited porosity and lower permeability, which may restrict the monomer penetration. In contrast, hardwoods such as alder and beech possess vessel elements and more open pore structures, facilitating better diffusion of the polymer. These structural differences likely influenced the density outcomes observed prior to polymerization. According to the ANOVA, the densities were significantly influenced by both the species and modification group, even before polymerization (*p* < 0.001 and *p* = 0.00154, respectively), with a statistically significant interaction (*p* = 0.04754). The post-treatment data should be interpreted by considering these variations in initial density across the species and groups. The pre-polymerization analysis was conducted with all samples grouped according to the intended modification type for consistency. In the subsequent figure, we show post-treatment values directly compared to pre-treatment values. Despite the wide range of post-treatment densities, both figures share the same categorical structure. The differences are visualized in Figure 1.

The table below shows further dimensional and mass measurements taken after polymerization. Depending on the species and formulation, all samples showed density increases ranging from approximately 24% to 80%. According to the ANOVA results, both the species (F(2, 108) = 426.827, *p* = 0.001) and modification type (F(3, 108) = 5.473, *p* = 0.00154) significantly influenced the post-treatment density, while there was a significant interaction effect (F(6, 108) = 2.209, *p* = 0.04754). The physical properties of the materials after polymerization are presented in Table 4.

**Figure 2:**
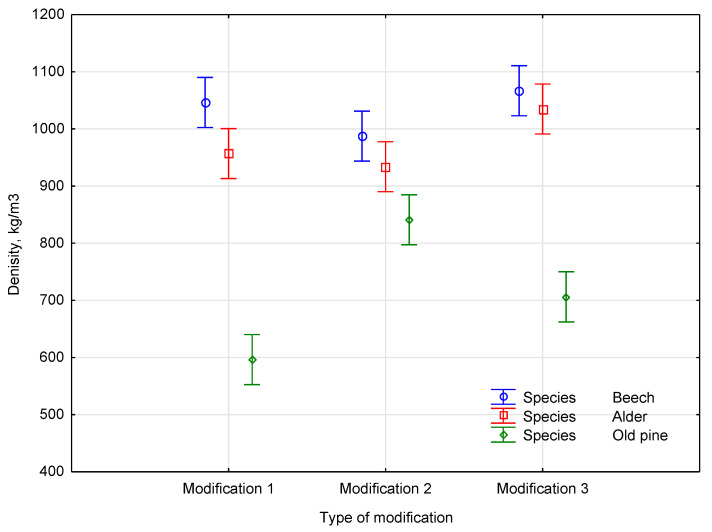
The impact of the material density after the polymerization of each species type.

All three species exhibited relative density increases, except for the alder, which demonstrated the greatest relative density increase, indicating higher monomer uptake. Despite the use of styrene in all formulations, the concentration of maleic anhydride may have influenced the effectiveness of the polymer–wood interaction. The degree to which the polymers integrated into and bonded with the wood matrix was likely affected by these differences. The statistical analysis produced homogeneous groups, from which the impact of the material density after the polymerization of each species type is presented in Figure 2.

The results of a two-way ANOVA revealed that both the wood species and modification type had significant effects on the density. The main effect of the species was highly substantial with *F*(2, 108) = 426.827 and *p* < 0.001, indicating substantial density variation among the three species. The main effect of the modification type was also significant, with *F*(3, 108) = 5.473 and *p* = 0.00154, confirming that the modifications significantly influenced the density. A statistically significant interaction between the species and modification type representing *F*(6, 108) = 2.209 and *p* = 0.04754 suggests that the modification’s effects on the density varied across species. The beech consistently exhibited the highest density across all modification types, while the old pine generally showed the lowest. The alder appeared to have high and very high-density ranges across the modifications. These observed trends are consistent with the significant interaction effect, suggesting varying modification effectiveness across species.

Different material structures were formed in each case based on the observed density differences among the polymerization formulations. Despite using the same monomer base (styrene), the presence of different maleic anhydride concentrations likely affected the degree of interaction between the polymer and the wood matrix. These chemical variations can affect the length of polymer chains, the crosslinking, and the adhesive bond with the lignin of a composite. Due to its anatomical structure and chemical affinity, alder exhibited the highest density increase across all formulations, suggesting greater monomer uptake and better polymer integration. The results show that both the wood species and the polymerization mixture’s chemical composition jointly influence the composite’s final density.

### 3.2. Analysis of Weight Percent Gain (WPG)

This study investigated the weight percent gain (WPG) of wood post-polymerization, a key indicator of treatment effectiveness. The WPG reflects the amount of polymer incorporated into the wood structure and can be determined by measuring the change in weight after treatment. Three wood species were treated with three different polymerizing mixtures, and the resulting WPG values were analyzed. The influence of the wood species and mixture composition on the WPG was assessed through a statistical analysis. Additionally, WPG was correlated with changes in wood density before and after modification. This research contributes to a deeper understanding of the mechanisms involved in wood polymerization. The wood density and WPG parameters for beech, alder, and pine are presented in Table 5.

**Figure 3:**
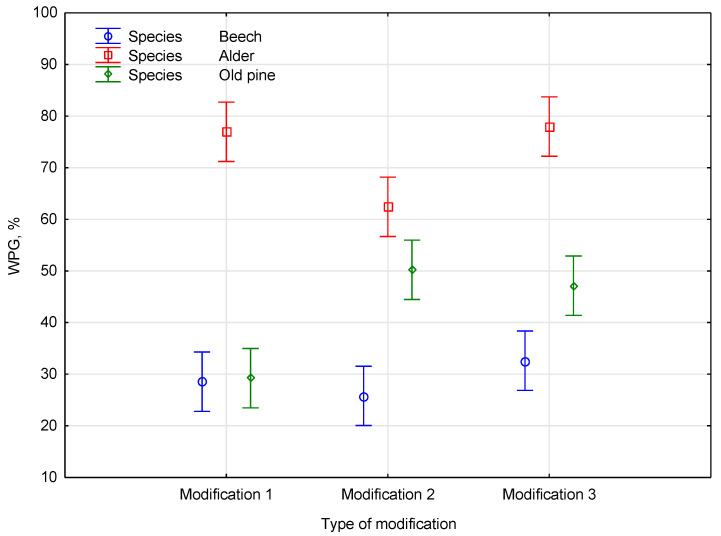
The impact of the weight percent gain (WPG) after the polymerization of the species type.

The statistical analysis revealed a highly significant main effect for the species with *F*(2, 81) = 178.157 and *p* < 0.001, indicating that the dependent variable varied substantially across the different wood species. This *p*-value of less than 0.001 signifies a strong statistical dependence between the species and the dependent variable. The main effect of the modification type was also statistically significant, with *F*(2, 81) = 6.073. The *p*-value of 0.00349 indicates a statistically significant relationship between the kind of modification and the dependent variable. Furthermore, a highly significant interaction was observed between the species and type of modification *F*(4, 81) = 9.869. The *p*-value of less than 0.001 for the interaction term shows a strong statistical dependence between the combined effects of the species and modification type and the dependent variable. The impact of the weight percent gain (WPG) after the polymerization of the species type is presented in Figure 3.

The results show that the relationship between the modification and WPG appears complex and species-dependent. While we propose that differences in anatomical structure and chemical composition, such as the pore size, lignin content, and presence of extractives, may contribute to variations in WPG, this explanation is not directly supported by the measurements in the present study. Neither the porosity nor the chemical composition was quantified using microscopic or spectroscopic methods but rather based on the literature data and general knowledge of the tested species. As such, the explanation remains hypothetical and should be validated in future studies through detailed anatomical and chemical analyses.

### 3.3. Analisis of Strength Tests

The mechanical properties of wood are critical for determining its suitability in various applications. Compression tests were performed to evaluate the effects of the modification process on these properties. The results from these tests are presented in this section, focusing on the average compressive strength (expressed as the pressure required to reduce a sample’s height by 5%). Comprehensive overviews of the compressive strength are provided for the beech, alder, and old pine samples subjected to various modification regimes. The standard deviations are included to represent the variability within each group. A post hoc analysis was subsequently performed to identify homogeneous groups, enabling a more detailed analysis of the relationships between the species, modification type, and compressive strength.

The role of polymerization in the mechanical performance was further investigated by examining the correlation between the physical parameters (density, WPG) and compressive strength. As a result, the samples with a greater weight-to-grain ratio and increased density displayed significantly improved compressive strength, especially in low-density woods such as old pine. The results of this study support the hypothesis that the structural stiffness is improved by greater monomer absorption. The average compressive strength values for beech, alder, and pine are presented in Table 6.

The wood density was significantly affected by the wood species and modification type in an analysis of variance. The species had a highly significant main effect, with *F*(2, 108) = 426.827 and a *p*-value of less than 0.001. Based on these results, the wood species showed significant differences in density. The main effect of the type of modification also proved statistically significant. In this case, *F*(3, 108) was 5.473, and the *p*-value was 0.00154. According to this result, the wood density is significantly affected by the modification type. An examination of the interaction between the species and type of modification was conducted. A significant interaction was found, with *F*(6, 108) = 2.209 and a *p*-value of 0.04754. The modifications to the density did not have the same effect for all wood species but did depend on the combination of species and changes.

Based on a statistical analysis, the wood species and modifications had varying effects on the compressive strength. The compressive strength of wood is affected by both the inherent properties and the specific modification process, emphasizing the need to consider both factors when optimizing the material. The different wood species, such as beech, alder, and pine, differed in their compressive strength values, illustrating inherent mechanical differences. The modification processes also significantly improved the compressive strength, indicating their potential for material improvement. These modifications were not effective across all species, suggesting species-specific responses. The wood modification process must be approached in a nuanced manner based on the wood’s specific properties. This study provides a deeper understanding of wood species, modification types, and compressive strength, giving insight into developing modified wood products with improved mechanical properties. The Young’s modulus values were measured for both the reference and modified samples. In all species but beech and alder, the polymerization caused increased stiffness. The in situ polymerization enhanced both the compression strength and elasticity. The values presented below were obtained from direct mechanical testing, including those for the reference group. All comparisons were based on the untreated samples. The Young’s modulus values for beech, alder, and pine are presented in Table 7.

The Young’s modulus values were measured for both the reference and modified samples to compare their stiffness before and after polymerization. In the testing of the wood samples, the polymerization significantly affected thier stiffness, as evidenced by the changes in Young’s modulus values. According to the modified samples, the beech exhibited the greatest stiffness increase, reaching 15.371 GPa vs. 11.001 GPa in the reference sample. The performance of the alder also improved, with the maximum value rising from 7.433 GPa in the reference to 11.156 GPa after modification. Comparatively, the 5.886 GPa value for the untreated old pine sample increased to 6.301 GPa after treatment. The correlation coefficients indicate that the modification process and changes in mechanical properties are closely related, with the values generally exceeding 0.80. According to these findings, polymerization can effectively enhance the structural performance of hardwoods such as beech and alder.

## 4. Discussion

The tested wood samples demonstrated significant improvements in their compressive strength after the in situ polymerization of Scots pine, European beech, and black alder. The increases in density, stiffness, and deformation resistance were due to the polymer filling the voids within the wood structure. The WPG, density gain, and compressive strength results strongly suggest that the polymeric material was incorporated into the wood structure and contributed to its mechanical reinforcement. However, in the absence of direct spectroscopic or microscopic confirmation, the existence of a true chemically bonded wood–polymer composite must be considered speculative. The current classification relies on performance-based indicators, as supported in similar studies [30,31], and should be interpreted as an operational rather than definitive definition. Future research studies will aim to validate the presence and distribution of the polymer through FTIR spectroscopy, SEM imaging, and µCT analyses. As a limitation of this study, neither spectroscopic nor microscopic techniques were applied to confirm the polymer distribution or bonding at the molecular level. According to this study, wood–polymer composites show increases in performance-based indicators such as the density, compressive strength, and weight percent gain (WPG), widely recognized as sufficient criteria for classifying wood as a composite material when there is no direct chemical evidence [30,31]. Spectroscopic or microscopic confirmation efforts would allow a practical assessment of the polymer integration effects, even without spectroscopy or microscopy. A chemical characterization of the polymer–wood interactions will be conducted in the future to provide a more complete understanding of the composite formation process.

There has been a lot of research on the effect of wood density on the effectiveness of a modification. The investigated [30,31] how the wood density and moisture content influence the mechanical characteristics of Coast Douglas-fir and found that density variations can significantly affect the wood properties [32]. Different anatomical structures and chemical compositions likely contributed to the differences in strength enhancement among the three species. The evidence for this is particularly evident when analyzing the density changes. The most pronounced increase was observed in the alder wood, perhaps due to its higher porosity and compatibility with the polymer formulations. The presence of the 2% and 3% maleic anhydride additions may have enhanced the grafting efficiency on the lignin, enhancing the polymer retention and density. The material performance is determined by the chemical formulation, which suggests that the formulation optimization should be species-specific. For instance, the polymerization may fill larger voids more effectively than in beech pine because pine is less dense. This is supported by studies such as that of Kamperidou (2021) [3], which examined the chemical and structural characteristics of poplar and black pine wood after thermal modification and found that variations in wood anatomy and composition can lead to different responses to modification treatments. The old pine samples assigned to modification 2 may have had higher variability due to natural heterogeneity in the aged wood [33].

The initial state of the material must be considered when discussing physical property changes. Since the pine started with the lowest density, it showed the largest increases in compressive strength and moderate density gains due to its greater porosity and capacity for monomer absorption. The initial density shifts in the beech were smaller, likely owing to its tighter cell structure limiting the penetration. The alder was especially noteworthy, despite its moderate initial density. It showed the greatest density gain, which may have resulted from its favorable porosity and compatibility with the polymer formulation. The maleic anhydride level was the main variable in all mixtures, even though the polymer densities were not measured independently. Studies should determine the polymer densities and perform microscopic analyses to link the porosity to the polymer distribution directly in the future.

Research has shown that various treatments enhance wood’s mechanical properties, consistent with the broader findings in wood modification research. In the literature studies [32] reviewed various functional treatments for modified wood, including polymer impregnation, and found significant improvements in strength and stiffness. The average compressive strength of the beech increased by 60% after polymerization, from 8.669 MPa to 13.869 MPa. There was a 119% increase in the average compressive strength of the alder from 5.644 MPa to 12.367 MPa. The average compressive strength of the pine increased by 150% from 4.519 MPa to 11.311 MPa. According to these results, polymer-modified wood has similar properties to other types of wood. One study found that polymerization increases the compressive strength of pine wood [24]. Wood species have different densities and structures, which may explain why their strength increases vary [32,34].

The least dense wood, pine, showed the highest percentage increase in strength, while the thickest wood, beech, showed the lowest. The increases in the Young’s modulus and WPG values correlated positively with the strength gains, suggesting greater structural stiffness and deeper polymer integration can explain the mechanical enhancements. The alder and beech achieved similar final compressive strength values after polymerization, at approximately 14 MPa and 12 MPa, respectively. The substantial differences in anatomical features and initial density make this result noteworthy. In both species, the polymer penetration may be even greater because they are diffuse–porous hardwoods. The WPG values for both indicate similar uptake behaviors. Polymer-based modification can consistently strengthen hardwoods when the vessel distribution is uniform. This may explain why both species performed similarly despite their structural differences.

The results of this study correspond to those of Mattos et al. (2015) [22], who showed significant increases in compressive strength after methacrylate monomer in situ polymerization [24]. Due to the increased strength, less dense wood species may benefit more from polymerization. The study findings show that modified wood can be used for various applications. The enhanced strength properties of polymer-modified timber make it an excellent alternative to traditional wood in construction, furniture, and other structural applications. This study focused on static compressive tests to assess the effects of polymerization on the mechanical properties of wood. Nevertheless, cyclic compressive tests could provide a more comprehensive understanding of these properties. Cycling tests, in particular, can provide information about the modified wood’s elastic region, energy loss, and damping capacity, which are all essential considerations for various applications. Using cyclic compressive tests might be an effective way of further examining changes in the wood’s viscoelastic behavior and energy dissipation capacity due to modification.

The results of this study are particularly relevant for industries wishing to recycle demolition timber into high-performance materials that are aligned with the circular economy concept. Wood that would otherwise be discarded shows increased compressive strength, especially when it is aged. This study aimed to evaluate polymer-modified wood’s mechanical performance, and more precisely its compressive strength. Chemical characterization and other physical analyses, along with µCT X-ray imaging, including FTIR, XRD, and cyclic mechanical testing, could provide further insight into the modification process and its results. Still, these methods were not within the scope of this study. This research can be continued to explore these aspects in depth to gain a more comprehensive understanding of the effects of polymerization on wood species in the future. Future research studies could explore the use of cyclic compressive tests to further investigate the effects of modification on the wood’s viscoelastic behavior and energy dissipation capacity. This would contribute to a more comprehensive understanding of the mechanical performance of modified wood under various loading conditions. The use of in situ polymerization to modify wood throughout its volume can contribute to sustainability initiatives by reducing the reliance on virgin wood sources and extending the service lives of timber products. Researchers should investigate polymer-modified wood’s long-term durability, dimensional stability, and decay resistance. Using recycled polymers for the polymerization could enhance the technology’s environmental benefits.

## 5. Conclusions

This study demonstrated mechanical effects consistent with polymer-treated wood. While no direct spectroscopic or structural data were collected, the observed behavior aligns with previously reported composite systems. The polymerization process led to significant increases in the compressive strength of Scots pine (150%), European beech (60%), and black alder (119%), demonstrating its potential for enhancing the mechanical properties of wood. The polymerization process significantly improved the compressive strength values of all three wood species, with the variations in strength enhancement likely influenced by their anatomical and chemical differences. If a polymer–wood composite had not formed, there would not have been such a significant increase in mass, particularly in the WPG (weight percentage gain) parameter. The observed mass growth strongly suggests that the polymer has successfully integrated with the wood structure, supporting the conclusion that the material obtained is indeed a polymer–wood composite. According to the study, in situ polymerization significantly enhances the compressive strength of wood, especially lower-density and aged wood types, bringing them on par with or making them better than unmodified high-density wood. The results highlight the role played by the wood structure in determining the effectiveness of the polymerization and suggest that species-specific properties should be considered in optimization strategies.

The findings of this study can be used to develop sustainable, high-performance wood products. The use of polymerized wood in structural applications might be able to reduce the reliance on traditional, non-modified wood by improving the mechanical properties. As a new contribution to the field, this study provides quantitative evidence of the influence of polymerization on wood strength, supporting its potential for industrial applications in the future. In order to determine polymer-modified wood’s practical viability, future research studies should evaluate its durability, dimensional stability, and decay resistance. Further research on the environmental impact of the process, including the possibility of using recycled polymers, could enhance its sustainability. Modification 3 achieved the highest overall strength and WPG values among the three polymerization systems tested. Accordingly, a high activator content may enhance the polymer bonding and reinforce low-density species better.

Further research is needed to include chemical and structural analyses to better correlate the composition with the mechanical performance. Research studies should also be conducted at structural scales, such as with full-size beams and reclaimed timber elements modified with polymerization. The application of such materials to circular construction and sustainable renovation practices would yield crucial insights into their applicability for structural reuse.

## Figures and Tables

**Table 1:** Compositions of polymerization mixtures.

Modification Type	Monomer	Activator	Initiator
Reference	X	X	X
1	Styrene	X	Benzoyl peroxide, approximately 1%
2	Styrene	Maleic anhydride, approximately 2%	Benzoyl peroxide, approximately 1%
3	Styrene	Maleic anhydride, approximately 3%	Benzoyl peroxide, approximately 1%

**Table 2:** The moisture contents of the materials before polymerization.

Species	Average Moisture (SD), %
Beech	8.97 (0.41) ^a^
Alder	9.76 (0.29) ^b^
Old pine	9.64 (0.24) ^b^

SD—standard deviation; ^a,b^—homogeneous group.

**Table 3:** Physical properties of the materials before polymerization.

Species	Modification Type	Length (SD), mm	Width (SD), mm	Thickness (SD), mm	Mass (SD), g	Density (SD), kg/m^3^
Beech	Reference	30.420 (0.123)	20.118 (0.174)	20.181 (0.089)	9.860 (0.184)	798.352 (12.796) ^a^
	1	30.437 (0.148)	20.195 (0.375)	20.333 (0.282)	9.972 (0.296)	797.939 (16.105) ^a^
	2	30.308 (0.079)	20.066 (0.135)	20.242 (0.329)	9.796 (0.245)	795.775 (14.891) ^a^
	3	30.341 (0.066)	20.13 (0.146)	20.127 (0.434)	9.722 (0.269)	790.821 (9.966) ^a^
Alder	Reference	30.398 (0.146)	19.899 (0.283)	20.083 (0.183)	6.599 (0.469)	542.995 (34.248) ^bc^
	1	30.449 (0.104)	20.228 (0.300)	20.273 (0.365)	6.686 (0.471)	535.632 (38.250) ^c^
	2	30.424 (0.130)	19.945 (0.282)	19.953 (0.295)	7.082 (0.499)	584.686 (36.524) ^b^
	3	30.419 (0.113)	20.032 (0.172)	20.015 (0.196)	7.015 (0.452)	574.917 (32.199) ^bc^
Old pine	Reference	30.423 (0.189)	19.790 (0.496)	19.582 (0.526)	5.432 (0.764)	461.207 (66.357) ^d^
	1	30.366 (0.142)	19.876 (0.480)	19.652 (0.618)	5.436 (0.849)	459.481 (78.935) ^d^
	2	30.394 (0.197)	19.819 (0.407)	19.281 (0.866)	6.389 (0.746)	550.361 (62.257) ^bc^
	3	30.117 (0.371)	19.793 (0.446)	19.900 (0.671)	5.756 (1.130)	485.543 (93.477) ^d^

SD—standard deviation; ^a,b,c,d^—homogeneous group.

**Table 4:** The physical properties of the materials after polymerization.

Species	Modification Type	Length (SD), mm	Width (SD), mm	Thickness (SD), mm	Mass (SD), g	Density (SD), kg/m^3^
Beech	Reference	X	X	X	X	X
	1	30.421 (0.158)	19.943 (0.445)	20.193 (0.239)	12.8174 (0.366)	1046.230 (11.353) ^ab^
	2	30.443 (0.119)	20.180 (0.158)	20.312 (0.324)	12.3205 (0.231)	987.402 (12.343) ^ac^
	3	30.322 (0.085)	19.953 (0.148)	19.971 (0.413)	12.8919 (0.387)	1066.798 (7.467) ^b^
Alder	Reference	X	X	X	X	X
	1	30.405 (0.092)	20.038 (0.360)	20.229 (0.371)	11.7892 (0.308)	956.833 (24.869) ^c^
	2	30.469 (0.123)	20.044 (0.310)	20.102 (0.330)	11.4647 (0.428)	933.842 (27.051) ^c^
	3	30.402 (0.115)	19.899 (0.142)	19.884 (0.191)	12.449 (0.248)	1034.882 (15.099) ^ab^
Old pine	Reference	X	X	X	X	X
	1	30.421 (0.162)	19.760 (0.470)	19.669 (0.624)	7.025 (1.200)	596.432 (115.624) ^d^
	2	30.434 (0.245)	19.588 (0.394)	19.234 (0.864)	9.609 (1.441)	840.831 (140.926) ^e^
	3	30.275 (0.491)	19.689 (0.433)	19.867 (0.662)	8.349 (1.031)	706.171 (91.323) ^f^

SD—standard deviation; ^a,b,c,d,e,f^—homogeneous group.

**Table 5:** Wood density and WPG parameters for beech, alder, and pine.

Species	Modification Type	Density Before Polymerization (SD), kg/m^3^	Density After Polymerization (SD), kg/m^3^	WPG, %
Beech	Reference	798.35 (12.80)	X	X
	1	797.94 (16.11)	1046.23 (11.35)	28.55 (1.98) ^a^
	2	795.77 (14.89)	987.40 (12.34)	25.80 (1.80) ^a^
	3	790.82 (9.97)	1066.80 (7.47)	32.61 (1.36) ^a^
Alder	Reference	542.99 (34.25)	X	X
	1	535.63 (38.25)	956.83 (24.87)	76.95 (10.59) ^b^
	2	584.69 (36.52)	933.84 (27.05)	62.43 (9.73) ^c^
	3	574.92 (32.20)	1034.88 (15.10)	77.98 (8.85) ^b^
Old pine	Reference	461.21 (66.36)	X	X
	1	459.48 (78.93)	596.43 (115.62)	29.23 (7.54) ^a^
	2	550.36 (62.26)	840.83 (140.93)	50.22 (11.24) ^d^
	3	485.54 (93.48)	706.17 (91.32)	47.13 (16.59) ^d^

SD—standard deviation; ^a,b,c,d^—homogeneous group.

**Table 6:** The average compressive strength values for beech, alder, and pine.

Species	Modification Type	Average Compressive Strength (SD), MPa
Beech	Reference	8.669 (0.410) ^a^
	1	12.950 (0.359) ^bc^
	2	13.136 (0.254) ^bc^
	3	13.869 (0.389) ^b^
Alder	Reference	5.644 (0.914) ^d^
	1	10.955 (0.951) ^e^
	2	11.475 (0.864) ^ef^
	3	12.367 (0.850) ^cf^
Old pine	Reference	4.519 (0.952) ^g^
	1	11.311 (2.593) ^ef^
	2	6.106 (1.126) ^d^
	3	7.289 (1.877) ^h^

SD—standard deviation; ^a,b,c,d,e,f,g,h^—homogeneous group.

**Table 7:** The average Young’s modulus values for beech, alder, and pine.

Species	Modification Type	Average Young Module, GPa	Determination Coefficient
Beech	Reference	11.001	0.865
	1	15.371	0.949
	2	10.430	0.825
	3	14.583	0.911
Alder	Reference	7.433	0.918
	1	10.418	0.874
	2	11.135	0.870
	3	11.156	0.845
Old pine	Reference	5.886	0.892
	1	6.301	0.783
	2	6.195	0.615
	3	6.152	0.701

## Data Availability

The original contributions presented in this study are included in the article. Further inquiries can be directed to the corresponding author.
